# Selective Attention Enhances Beta-Band Cortical Oscillation to Speech under “Cocktail-Party” Listening Conditions

**DOI:** 10.3389/fnhum.2017.00034

**Published:** 2017-02-10

**Authors:** Yayue Gao, Qian Wang, Yu Ding, Changming Wang, Haifeng Li, Xihong Wu, Tianshu Qu, Liang Li

**Affiliations:** ^1^Beijing Key Laboratory of Behavior and Mental Health, School of Psychological and Cognitive Sciences, Peking UniversityBeijing, China; ^2^Beijing Anding Hospital, Capital Medical UniversityBeijing, China; ^3^Beijing Institute for Brain Disorders, Capital Medical UniversityBeijing, China; ^4^School of Computer Science and Technology, Harbin Institute of TechnologyHarbin, China; ^5^Department of Machine Intelligence, Peking UniversityBeijing, China; ^6^Key Laboratory on Machine Perception – Ministry of Education, Speech and Hearing Research Center, Peking UniversityBeijing, China

**Keywords:** selective attention, speech unmasking, long-term neural activities, neural network, motor theory, informational masking

## Abstract

Human listeners are able to selectively attend to target speech in a noisy environment with multiple-people talking. Using recordings of scalp electroencephalogram (EEG), this study investigated how selective attention facilitates the cortical representation of target speech under a simulated “cocktail-party” listening condition with speech-on-speech masking. The result shows that the cortical representation of target-speech signals under the multiple-people talking condition was specifically improved by selective attention relative to the non-selective-attention listening condition, and the beta-band activity was most strongly modulated by selective attention. Moreover, measured with the Granger Causality value, selective attention to the single target speech in the mixed-speech complex enhanced the following four causal connectivities for the beta-band oscillation: the ones (1) from site FT7 to the right motor area, (2) from the left frontal area to the right motor area, (3) from the central frontal area to the right motor area, and (4) from the central frontal area to the right frontal area. However, the selective-attention-induced change in beta-band causal connectivity from the central frontal area to the right motor area, but not other beta-band causal connectivities, was significantly correlated with the selective-attention-induced change in the cortical beta-band representation of target speech. These findings suggest that under the “cocktail-party” listening condition, the beta-band oscillation in EEGs to target speech is specifically facilitated by selective attention to the target speech that is embedded in the mixed-speech complex. The selective attention-induced unmasking of target speech may be associated with the improved beta-band functional connectivity from the central frontal area to the right motor area, suggesting a top-down attentional modulation of the speech-motor process.

## Introduction

The “cocktail-party” problem (Cherry, 1953) indicates the astonishing ability of human listeners to recognize target speech in noisy environments with multiple-people talking. It has been confirmed that selective attention plays a critical role in this perceptual/cognitive capacity (e.g., Brungart, 2001; Freyman et al., 2001, 2004; Roman et al., 2003; Li et al., 2004; Bidet-Caulet et al., 2007; Ezzatian et al., 2011; Golumbic et al., 2012; Mesgarani and Chang, 2012). On the other hand, non-selective attention provides more generalized and sustain alertness for preparing the emergence of high-priority signals (Posner and Petersen, 1990, 2012). The relationship between selective attention and non-selective attention has been an attractive issue in the visual research field (e.g., Coull et al., 1998; Matthias et al., 2010), but has not been systematically investigated in the auditory research field.

Recently, a few studies on how selective attention affects the cortical representation of target speech have been reported (e.g., Lalor and Foxe, 2010; Ding and Simon, 2012, 2013; Golumbic et al., 2013; Kong et al., 2014). Particularly, under “cocktail-party” listening conditions, selective attention modulates low-frequency oscillations of cortical responses to speech stimuli, exhibiting both enhanced tracking of target-speech signals and enhanced suppression of masker-speech signals (Kerlin et al., 2010; Lalor and Foxe, 2010; Power et al., 2010, 2012; Mesgarani and Chang, 2012; O’Sullivan et al., 2014). It is of interest to know how the neural representation of speech signals under “cocktail-party” conditions is affected by shifting non-selective attention to selective attention.

It has been proposed that low-frequency (alpha and beta bands) oscillations of cortical activation mainly carries top-down modulation information, while high-frequency (gamma) oscillations mainly carries bottom-up information (Wang, 2010; Bastos et al., 2012; Weiss and Mueller, 2012; Bressler and Richter, 2015; Friston et al., 2015; Lewis and Bastiaansen, 2015). Particularly, top-down signals that come to lower-level brain structure underlies the attentional processing that is associated with the synchrony in the beta frequency band (Hanslmayr et al., 2007; Womelsdorf and Fries, 2007; Donner and Siegel, 2011; Bressler and Richter, 2015; Saarinen et al., 2015; Todorovic et al., 2015). More specifically, for example, beta-band activity is related to various top-down cognitive/perceptual processes (review in Engel and Fries, 2010), including prediction (Engel et al., 2001; Ahveninen et al., 2013; Todorovic et al., 2015; Lewis et al., 2016) and motor control (Brittain and Brown, 2014; Piai et al., 2015). Also, the beta-band oscillation represents functional connectivity between the frontal cortex and motor cortex in attention tasks (Thorpe et al., 2012; Piai et al., 2015). It is of interest to know whether neural oscillations in the beta band are involved in speech unmasking based on selective attention.

The present study investigated whether neural oscillations of scalp-recoded electroencephalogram (EEGs) to multiple-talker (voice) speech are modulated by selective attention and what are the potential underlying mechanisms. EEG signals were recorded from participants who either selectively attended to one of the talker’s voice or non-selectively attended to the whole mixed-speech complex. Four frequency bands (theta: 4–8 Hz; alpha: 8–12 Hz; beta: 13–30 Hz; gamma: 30–48 Hz) of recorded EEGs were analyzed to reveal both the cortical representation of speech signals and the differences in cortical causal connections between the selective attention condition and the non-selective attention condition. Across EEG correlations were used to estimate whether the cortical speech representation becomes more correlated to the attended target speech under the selective attention condition than the non-selective attention condition.

## Materials and Methods

### Participants

Twelve younger adults (five males and seven females) with the mean age of 23.6 years old (from 19 to 25 years old) were recruited from Peking University as the participants in this study. They provided informed consent to participate in this study and were paid a modest stipend for their participation. All the participants were right-handed native Mandarin Chinese speakers with normal and balanced (no more than 15 dB difference between the two ears) pure-tone hearing thresholds between 125 and 8000 Hz. The participants gave their written informed consent for participation in this study. The experimental procedures used in this study were approved by the Committee for Protecting Human and Animal Subjects of the Department of Psychology at Peking University.

### Speech Stimuli

The speech stimuli used in this study were Chinese “nonsense” sentences. “Nonsense” sentences are syntactically correct but not semantically meaningful (e.g., Freyman et al., 1999; Li et al., 2004; Yang et al., 2007; Gao et al., 2014). Direct English translations of these Chinese sentences are similar but not identical to the English “nonsense” sentences used in previous studies (Helfer, 1997; Freyman et al., 1999, 2004; Li et al., 2004). For example, the English translation of one Chinese nonsense sentence is “That corona removes the crest-span bag”. The development of the Chinese “nonsense” sentences has been described elsewhere (Yang et al., 2007).

**FIGURE 1:**
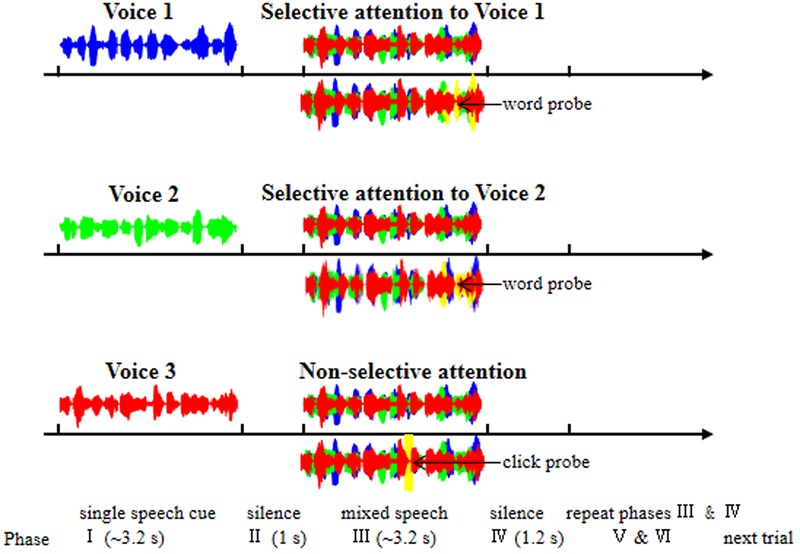
**Illustration of the six phases within each trial of EEG recordings.** Phase I: a trial was started with the presentation of a single-voiced speech (Voice 1, 2, or 3) to indicate which stimulation condition the present trial belonged to [Voice 1, selective attention to Voice 1 (top panel); Voice 2, selective attention to Voice 2 (middle panel); Voice 3, non-selective attention to the whole mixed-speech complex (bottom panel)]. Phase II: a period of silence lasting 1 s. Phase III: the presentation of the mixed three-voiced speech. Phase IV: a period of silence lasting 1.2 s. Phase V and Phase VI: the repetition of Phase III and Phase IV, respectively. Under the selective-attention condition (with Voice 1 or 2), participants were instructed to press a button if they had heard a wrong words probe (yellow waves); under the non-selective-attention condition, participants were instructed to press a button if they heard a click probe (yellow waves). The blue, green, and red waves indicate the single speech of Voice 1, Voice 2, and Voice 3, respectively.

In this study, three different younger-adult female talkers recited the speech stimuli with different sentences. In a typical recording trial, during the mixed-speech presentation when EEGs were recorded (Phase III in **Figure 1**), the three voices reciting differences sentences were presented at the same time, simulating a “cocktail-party” listening condition. Before the 3-voice mixed-speech presentation, one of the speech stimuli was presented alone (Phase I in **Figure 1**) to indicate that either the repeatedly presented speech in the mixed-speech presentation was the target speech when the pre-presented speech was recited by Voice 1 or 2, or there was no particular (single) target speech in the mixed-speech presentation when the pre-presented speech was recited by Voice 3. Consequently, the target speech was determined (when recited by Voice 1 or 2), and the other two speech stimuli formed the masker. In other words, the target speech was presented against a two-talker-speech background. Note that two-talker speech maskers were the most effective in inducing informational masking (Freyman et al., 2004). Each of the three voices recited different sentences and the sound pressure level of the three voices were the same. The mean duration of the sentences was 3.26 s (ranged from 3.1 to 3.5 s).

All speech signals were digitized at a sampling rate of 22.05 kHz using a 24-bit Creative Sound Blaster PCI128 with a built-in anti-aliasing filter (Creative Technology, Ltd., Singapore). All the stimuli, including the single-voice speech, mixed-voice speech, and click sounds were transferred using a Creative Extigy sound blaster and presented to participants at the two ears without any interaural time disparities using two tube-ear inserts (Neuroscan, El Paso, TX, USA). The sound pressure level of a single voice was set at 56 dB SPL, calibrated by a Larson Davis Audiometer Calibration and Electroacoustic Testing System (Audit and System 824, Larson Davis, USA). Since the sound pressure level of the three voices were the same, the signal-to-masker ratio (SMR) was -3 dB when a target speech was determined in the mixed-speech presentation.

### Electrophysiological Recordings

Scalp EEG recordings (with the reference electrode located on the nose) were conducted in a dim double-walled sound-attenuating booth (EMI Shielded Audiometric Examination Acoustic Suite) that was equipped with a 64-channel NeuroScan SynAmps System (Compumedics Limited, Abbotsford, VIC, Australia). EEG signals were processed with a sample rate of 1000 Hz, on-line amplified 500 times, and low-pass filtered below 200 Hz. Eye movements and eye blinks were recorded from electrodes superior and inferior to the left eye and also at the outer canthi of the two eyes. The impedances of all the recording electrodes were kept below 5 kΩ.

### Procedures

The effect of selective attention was estimated by examining the differences in EEGs between the selective attention condition and the non-selective attention condition. Voice 1 and Voice 2 were used as either the target voice or the masking voice, and Voice 3 was used only as the masking voice. There were three stimulation conditions for the mixed-speech presentation: (1) Condition 1: selective attention only to Voice 1, (2) Condition 2: selective attention only to Voice 2, and (3) Condition 3: non-selective attention to the whole speech complex (**Figure 1**).

In addition to the 3-voice mixed-speech presentation, each of the speech stimuli was presented alone to obtain EEGs to the single-speech presentation.

In this study, five “nonsense” sentences from a pool with totally 360 sentences were randomly assigned to a participants (two sentences for Voice 1; other two different sentences for Voice 2; one sentence for Voice 3) and different participants listened to difference sentences. For each participants, there were four different mixed-speech presentations.

As shown in **Figure 1**, each trial contained six phases: In Phase I, a trial was started with the presentation of a single-voiced speech (Voice 1, 2, or 3) with the duration about 3.2 s as the cue to indicate which stimulation condition the present trial belonged to (Voice 1, Condition 1; Voice 2, Condition 2; Voice 3, Condition 3). Phase I was followed by Phase II, which was a period of silence lasting 1 s.

In Phase III, the mixed three-voiced speech (about 3.2 s) was presented (the same stimuli under different conditions for a participant). Phase IV was also a period of silence lasting 1.2 s. The Phase V and Phase VI were the repetition of Phase III and Phase IV, respectively. In other words, the mixed speech presentation occurred twice in a trial.

Under a selective attention condition (Condition 1 or 2), participants were instructed to pay attention to the target voice and press a button if they had heard a novel “predicate-object” structure presented with the same voice as the to-be-attended talker (as the false-word probe, with four syllables and the possibility of 14.2%, **Figure 1**). Under non-selective attention condition (Condition 3), the participants were instructed to pay attention to the whole speech complex and press a button if they heard a “click” (as the probe with the possibility of 14.2%) at a random time position (**Figure 1**). To ensure that the participants could understand and follow the instructions, a training session was conducted before EEG recordings. The percent correct in detecting the probe in each of the participants were required to be no less than 85%.

In total, there were 96 stimulation presentations for EEG recordings (after the removal of the presentations with probes) for each of the three conditions, and these 96 presentations were randomly assigned into four blocks. Each block contained 24 stimulation presentations for each of the three conditions whose presenting order was arranged randomly for a participant. It took about 10 mins to complete one block. To limit eye movements, participants were also asked to stare a cross in the front in a trial.

### Data Analyses

Using the EEGLAB toolbox (Delorme and Makeig, 2004) in MATLAB, raw EEG data were filtered by three different band-pass filters (alpha: 8–12 Hz; beta: 12–30 Hz; gamma: 30–48 Hz), and then segmented into epochs from -300 to 3500 ms relative to the onset of a mixed-speech presentation. The baseline correction was conducted in the period of -300 to 0 ms before the presentation onset. The epochs that contained more than ±30 μV potential were rejected as artifacts. The rest of epochs were averaged for each condition to analyze the grange causality and across EEG correlations.

To avoid the onset and offset (above 3000-ms) effect (Pasley et al., 2012), the period of interest was defined within the time 800–2800 ms after the mixed-speech presentation onset. The across EEG correlation was calculated by the *corr* function in MATLAB. The Granger Causality (GC) analysis was calculated using the Brainstorm toolbox (Tadel et al., 2011)^1^ in the MATLAB environment to estimate causal connectivity associated with the selective attention effect.

Six areas were defined for GC analyses: (1) the left frontal area, including sites F5, F3, F1, FC5, FC3, FC1; (2) the central frontal area including sites, including F3, F1, F2, FC3, FC1, FC2; (3) the right frontal area, including sites F6, F4, F2, FC6, FC4, FC2; (4) the left motor area, including sites C5, C3, C1, CP5, CP3, CP1; (5) the central motor area, including sites C3, C1, C2, CP3, CP1, CP2; (6) the right motor area, including sites C6, C4, C2, CP6, CP4, CP2. The areal GC value for each participant was averaged by the GC values of all site connections in each area.

The change index was calculated as: (v1 - v2)/(v1 + v2), where the v1 and v2 were the value under two different conditions.

Statistical analyses were performed with IBM SPSS Statistics 20 (SPSS Inc., Chicago, IL, USA). Within-participants, paired *t*-tests and Pearson correlation were conducted to assess differences between conditions. The null-hypothesis rejection level was set at 0.05.

## Results

### The Effect of Selective Attention on Cortical Representations of Speech Signals against Speech Masking

To estimate the effect of selective attention on cortical representation of speech against speech masking, Pearson correlation coefficients were calculated between the EEGs to the mixed-speech complex under the selective attention condition (when only one voice was attended) and the EEGs to a single-voiced speech, which was used as either the attended one (the target voice) or not the attended one in the mixed-speech complex.

**FIGURE 2:**
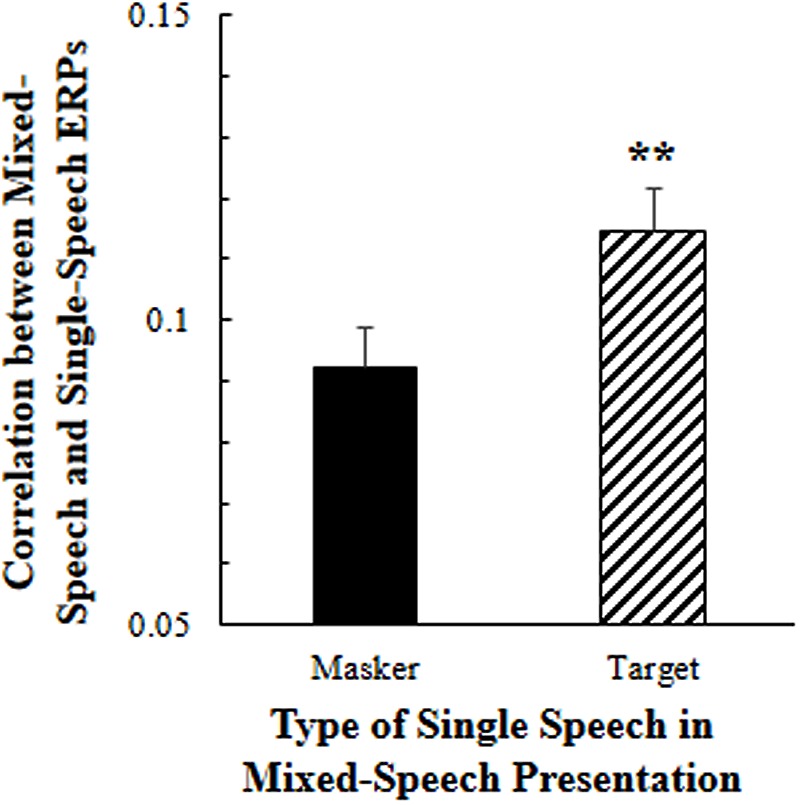
**Under the selective attention condition, the correlation between the all-site-averaged EEGs to the mixed-speech complex and the all-site-averaged EEGs to the single speech that was either the target or the masker speech in the mixed-speech complex.** ^∗∗^*p* < 0.01, paired *t*-test. The error bar indicates the standard errors of the mean.

As showed in **Figure 2**, the 5-Hz high-pass filtered all-site-averaged EEGs to the mixed-speech complex were significantly more correlated to the 5-Hz high-pass filtered all-site-averaged ERPs to the single speech that was used as the target speech in the speech complex than the EEGs to the single speech that was not attended in the speech complex [*t*(11) = 3.124, *p* = 0.010, paired *t*-test]. These results suggested that selective attention significantly improved the cortical representation of target-speech signals in a multi-talker environment (Supplementary Figure S1).

**FIGURE 3:**
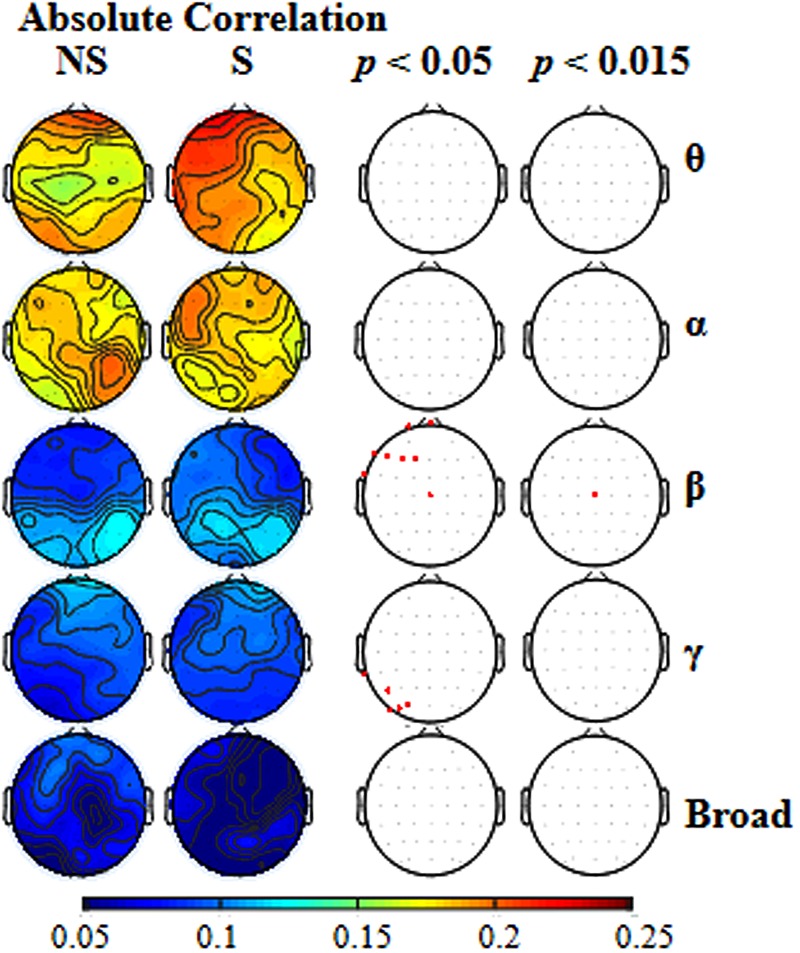
**The two left columns: for each of the five types of frequency bands [theta (𝜃), alpha (α), beta (β), gamma (γ), broad], the scalp topographical maps showing location distributions of absolute correlations between the EEGs to the mixed-speech complex and EEGs to a single speech under either the non-selective attention (NS) condition or the selective attention (S) condition.** The two right columns: for each of the frequency bands, the recordings sites at which the correlation difference between the two attention conditions was significant when the *p* level was either 0.05 and or 0.015.

To further estimate whether different frequency-band oscillations in EEGs were differently affected by selective attention, EEG data for each of the various frequency bands (theta, alpha, beta, gamma, and broad) were analyzed separately. In **Figure 3**, for each of the frequency bands, the first left column shows the absolute correlation coefficients between the EEGs to the mixed-speech complex under the non-selective attention condition (NS) and the EEGs to a single speech for all the recording sites; the second left column shows the absolute correlation coefficients between the EEGs to the mixed speech under the selective attention condition (S) and the EEGs to the target single speech for all the recording sites.

To reveal the frequency band that was the most vulnerable to selective attention, **Figure 3** also shows the statistically thresholded topographical map (the two right columns) indicating the electrode sites exhibiting significant differences in absolute correlation coefficient between the selective attention condition (S) and the non-selective attention condition (NS). When the *p* level was 0.05 (the second right column), both beta- and gamma-band components of EEGs recorded from a few electrode sites exhibited significant differences between the two attention conditions. **Table 1** shows the *p*-values for these electrode sites. Also shown in **Table 1**, only the beta-band component of EEGs recorded from the site Cz exhibited a significant difference between the two attentional conditions when the *p* was as low as 0.011. In other words, the beta-band obtained at the site Cz was the only component exhibiting a significant difference between the two attention conditions when the *p* value was less than 0.020. The right column in **Figure 3** presents the results indicating that the beta-band component of EEGs at the site Cz was the only one exhibiting a significant difference between the two attention conditions when the *p* value was 0.015 (which was just larger than 0.011 but smaller than 0.020). More in detail, at the *p* level of 0.015, the mixed-speech-evoked EEGs at site Cz were significantly more correlated with the single-speech-evoked EEGs under the selective attention condition than under the non-selective attention condition for beta band [*t*(11) = 3.029, *p* = 0.011, paired *t*-test], but not for other bands (both *p* > 0.05, paired *t*-test), indicating that the EEG beta-band component at the site Cz was the most vulnerable to selective attention (Supplementary Figure S2).

**Table 1:** Electrode sites at which beta and gamma bands were significantly different between the two attention conditions.

Band	Sites	*df*	*t*	*p*
Beta	CZ	11	3.029	0.011
Beta	F7	11	2.642	0.023
Beta	F1	11	2.494	0.030
Beta	FT7	11	2.408	0.035
Beta	F3	11	2.384	0.036
Beta	FP1	11	2.325	0.040
Beta	FPZ	11	2.258	0.045
Beta	F5	11	2.203	0.050
Gamma	PO7	11	2.593	0.025
Gamma	PO5	11	2.554	0.027
Gamma	P5	11	2.372	0.037
Gamma	TP7	11	2.311	0.041
Gamma	PO3	11	2.283	0.043

### Beta-Band Causal Connectivity Enhanced by Selective Attention

**FIGURE 4:**
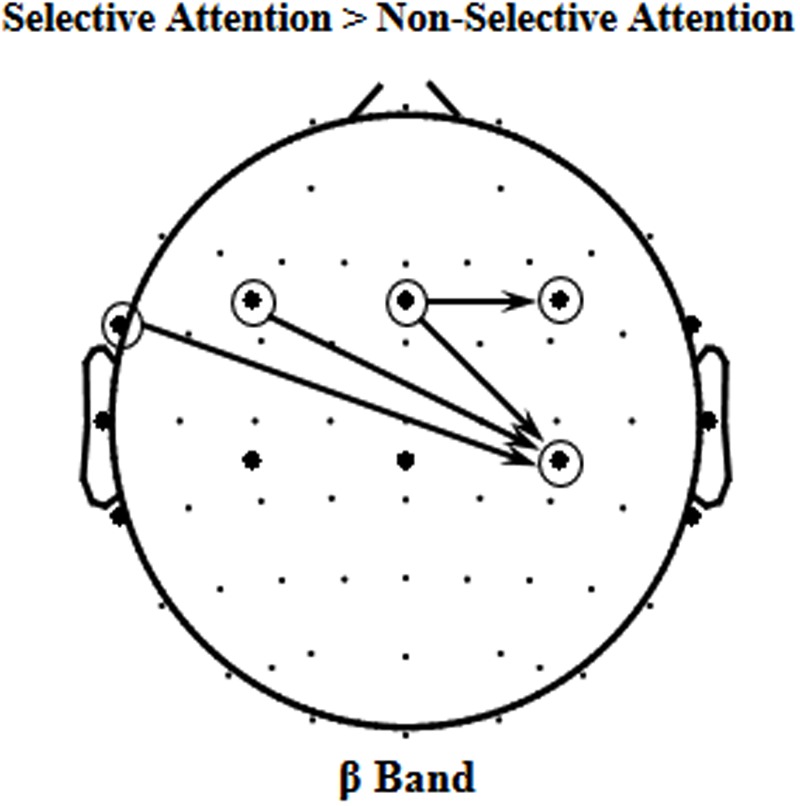
**The four significant event-related Granger Causalities (GCs) induced by selective attention (S) against non-selective attention (NS) of beta band (*p* < 0.05)**.

Since the beta-band component in EEGs to speech was significantly enhanced by selective attention, it is of importance to know whether some causal connectivities (i.e., GCs) of beta band were also enhanced by selective attention. The results of GC analyses showed that the following four beta-band GCs were significantly facilitated by selective attention (*p* < 0.05, paired *t*-test, **Figure 4**), including the ones (1) from site FT7 to the right motor area [Voice 1, *t*(11) = 2.769, *p* = 0.018; Voice 2, *t*(11) = 2.371, *p* = 0.037], (2) from the left frontal area to the right motor area [Voice 1, *t*(11) = 3.223, *p* = 0.008; Voice 2, *t*(11) = 2.629, *p* = 0.023], (3) from the central frontal area to the right motor area [Voice 1, *t*(11) = 2.344, *p* = 0.039; Voice 2, *t*(11) = 2.451, *p* = 0.032], and (4) from the central frontal area to the right frontal area [Voice 1, *t*(11) = 3.895, *p* = 0.002; Voice 2, *t*(11) = 2.692, *p* = 0.021].

### Correlation between Causal Connectivity and Cortical Representation of Speech against Speech Masking

As described above, selective attention enhanced both the beta-band component of the cortical representation of the target speech in mixed-speech complex and the four GCs (the ones from site FT7 to the right motor area, from the left frontal area to the right motor area, from the central frontal area to the right motor area, from the central frontal area to the right frontal area). Thus, it is of interest to know whether the selective attention-induced beta-band improvement of the speech representation (measured by the correlation change index, see below) was correlated with the selective-attention-induced improvement of any of the four GCs (measured by the GC change index, see below).

The correlation change index induced by selective attention was calculated as: (ρ_S__-_ ρ_NS_)/(ρ_S_ + ρ_NS_), where ρ_S_ and ρ_NS_ were the beta-band EEG correlation coefficients between mixed-speech stimulation and single-speech stimulation at site Cz under the selective attention condition (S) and under the non-selective attention condition (NS), respectively. The positive value represented a selective attentional predominance while the negative value represented a non-selective attentional predominance.

The GC change index for a frequency band (such as beta band) was calculated as: (G_S,c__-_ G_NS,c_)/(G_S,c_ + G_NS,c_), where G_S_ and G_NS_ were the Granger Causalities for a connection *c* under the selective (S) attention condition and the non-selective (NS) attention condition, respectively.

**FIGURE 5:**
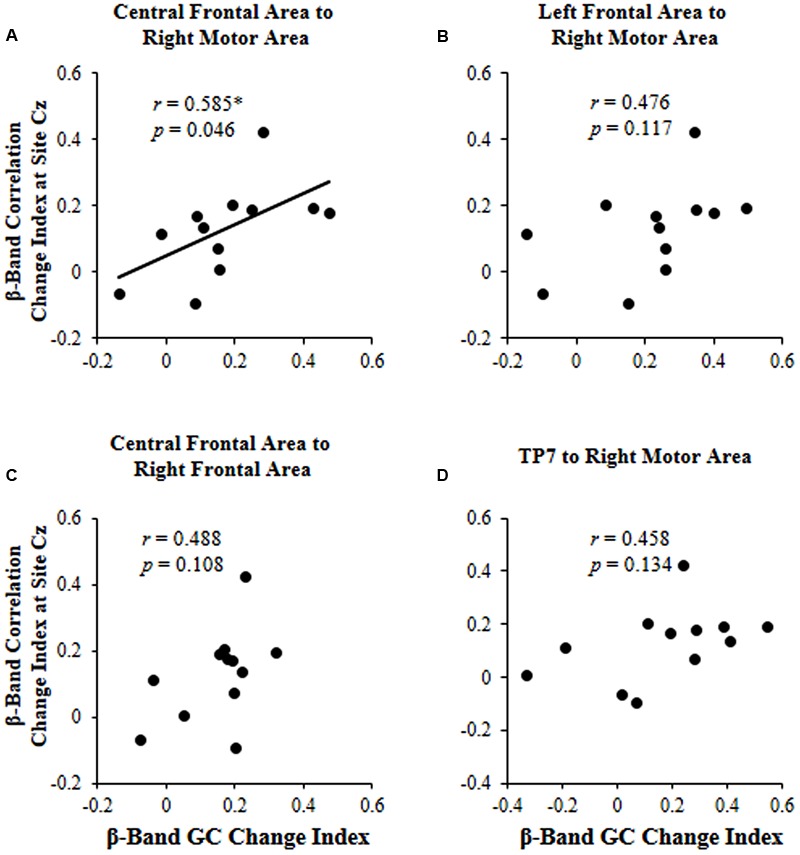
**For each of the four significant GCs shown in **Figure 4**, the correlation between the beta (β)-band correlation change index induced by selective attention and the beta (β)-band GC change index induced by selective attention. (A)** Causal connectivity from the central frontal area to the right motor area; **(B)** Causal connectivity from the left frontal area to the right motor area; **(C)** Causal connectivity from the central frontal area to the right frontal area; **(D)** Causal connectivity from site TP7 to the right motor area. ^∗^*p* < 0.05.

The results showed that the correlation change index of beta band across participants was significantly correlated with the beta-band GC change index only for connectivity from the central frontal area to the right motor area (*r* = 0.585, *p* = 0.046; **Figure 5**).

## Discussion

By recording scalp EEGs to speech stimuli, this study investigated under a simulated “cocktail” party condition with speech-on-speech masking, how selective attention modulates cortical representation of the masked target speech. Note that these findings on the difference between selective-attention and non-selective-attention conditions are based on the use of speech sounds. It is of importance to know whether similar findings can be obtained using non-speech sounds.

### Selective Attention Improves the Cortical Representation of Target-Speech Signals

Previous studies have reported that human cortical oscillations represent temporal structures of speech signals with high fidelity (Ding and Simon, 2012; Mesgarani and Chang, 2012). The results of this study showed that the correlation between the all-site-averaged EEGs to the mixed-speech complex and the all-site-averaged EEGs to the single speech that was used as the target in the speech complex was significantly larger than the correlation between the all-site-averaged EEGs to the mixed-speech complex and the all-site-averaged EEGs to the single speech that was not attended in the speech complex. Thus, this study supports the view that under a speech-on-speech masking condition, selective attention to a single-voice speech improves the cortical representation of this target single-voice speech (Ding and Simon, 2012, 2013; Mesgarani and Chang, 2012; Golumbic et al., 2013; O’Sullivan et al., 2014).

### The Beta-Band Component of the EEGs to Speech Is the Most Vulnerable to Selective Attention

In this study, following EEG data for each of the three frequency bands (alpha, beta, and gamma) were analyzed separately, the results showed that the beta-band component, but not either the alpha-band component or the gamma-band component, in the mixed-speech-evoked EEGs, was significantly more correlated with the single-speech-evoked EEGs under the selective attention condition (where the target single-voice speech was attended) than under the non-selective attention condition. Thus, the EEG beta-band component was the most vulnerable to selective attention.

Beta oscillations are associated with attention and predictions (Engel and Fries, 2010; Donner and Siegel, 2011; Weiss and Mueller, 2012; Todorovic et al., 2015), which are critical to speech cognition. Particularly, the top-down propagation of predictions reflected by beta oscillations (Engel et al., 2001; Bastos et al., 2012; Ahveninen et al., 2013; Lewis and Bastiaansen, 2015; Todorovic et al., 2015; Lewis et al., 2016) may be more critical for selective-attention-induced unmasking of speech, probably through enhancing the mechanism underlying binding distributed sets of neurons into a coherent representation of speech contents (Weiss and Mueller, 2012).

### Selective-Attention Facilitated Beta-Band Causal Connectivity from the Central Frontal Area to the Right Motor Area

The results of this study also showed that in total four beta-band causal connectivities (measured as GCs) were enhanced by selective attention, including the ones (1) from site FT7 to the right motor area, (2) from the left frontal area to the right motor area, (3) from the central frontal area to the right motor area, and (4) from the central frontal area to the right frontal area. However, only the selective-attention-induced enhancement of beta-band GC from the central frontal area to the right motor area was significantly correlated to the selective-attention-induced enhancement of the correlation between beta-band oscillations to the mixed speech complex and beta-band oscillations to the single speech. The results suggest that the selective-attention-induced improvement of beta-band representation of target speech signals is associated with the enhanced top-down modulation of the motor areas in the right hemisphere by the central frontal cortical areas. In other words, selective attention improves speech-related motor processes. However, due to the low spatial resolution of EEGs, whether the beta activities over central areas are based on the auditory or motor activity need further investigation in the future.

The *Motor Theory* of speech perception proposes that the interaction between the auditory and motor systems plays an essential role in speech perception (Liberman et al., 1952, 1967; Liberman and Mattingly, 1985; for review see Wu et al., 2014). It has been evident that speech perception activates the motor cortex (Fadiga et al., 2002; Callan et al., 2004; Wilson et al., 2004; Pulvermüller et al., 2006; Wilson and Iacoboni, 2006; Meister et al., 2007; Bever and Poeppel, 2010; Hickok et al., 2011; Elemans et al., 2015). Thus, under adverse listening conditions (such as the cocktail-party environment) where the perceptual load is high (Hickok and Poeppel, 2007; Fridriksson et al., 2008; Bishop and Miller, 2009), with the involvement of the motor system the listener can better identify the speaker’s intention and follow the target stream (Wu et al., 2014).

## Conclusion

(1) The cortical representation of target-speech signals under the multiple-people talking condition is specifically improved by selective attention, and the beta-band EEG component is the most vulnerable to selective attention.
(2) The selective-attention-induced enhancement of beta-band causal connectivity from the central frontal area to the right motor area is correlated with the selective-attention-induced enhancement of the cortical beta-band representation of target speech.
(3) Selective attention to a single-voiced target speech, which is embedded in a mixed-speech complex (with speech-on-speech masking), improves the cortical representation of the target speech by facilitating the top-down frontal modulation of the motor cortical areas.
(4) The unmasking of target speech based on selective attention may be caused by top-down attentional modulation of the speech-motor interactions.

## Author Contributions

YG, QW, and YD: Experimental design, experiment set up, experiment conduction, data analyses, figure/table construction, and paper writing. CW and HL: Experimental design, data analyses, and paper writing. XW: Experimental design and paper writing. LL: Experimental design, figure/table construction, and paper writing. TQ: Experimental design, experiment set up, and paper writing.

## Conflict of Interest Statement

The authors declare that the research was conducted in the absence of any commercial or financial relationships that could be construed as a potential conflict of interest.

## Footnotes

1 http://neuroimage.usc.edu/brainstorm/
