# Supplementary material for: Selective Attention Enhances Beta-Band Cortical Oscillation to Speech under “Cocktail-Party” Listening Conditions
Source: Front Hum Neurosci. 2017 Feb 10;11:34. doi: 10.3389/fnhum.2017.00034 (PMC5300994; doi:10.3389/fnhum.2017.00034)
Supplement: Supplementary file 1 [file Image_1.pdf]

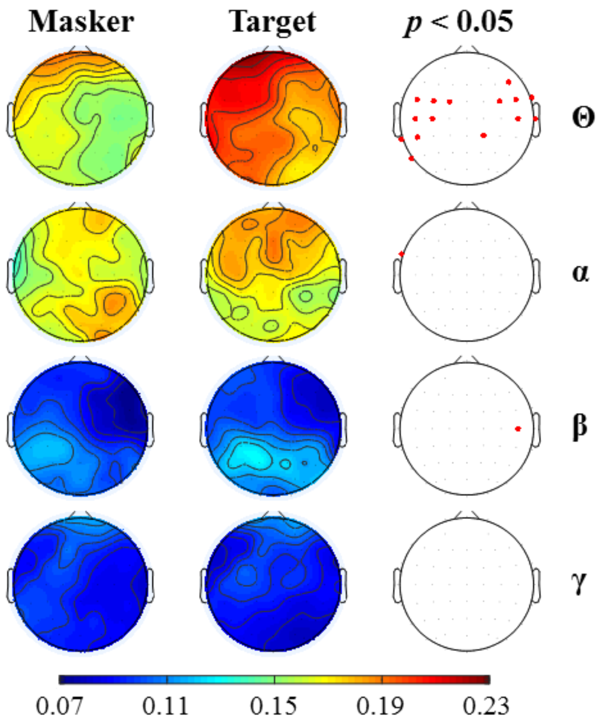

Figure S1. Scalp topographical maps showing location distributions of absolute correlations between the EEGs to the mixed-speech complex and the EEGs to either a single-voiced masker speech (left column) or a single-voiced target speech (middle column) for each of the 4 frequency bands [theta (θ), alpha (α), beta (β), gamma (γ)]. The right columns: for each of the 4 frequency bands, the recordings sites at which the correlation difference between the 2 correlations was significant when the p level was 0.05. The statistically thresholded topographical map indicating that the theta oscillation significantly tracked the target against the masker in the bilateral temporal cortex.
