# Supplementary material for: Selective Attention Enhances Beta-Band Cortical Oscillation to Speech under “Cocktail-Party” Listening Conditions
Source: Front Hum Neurosci. 2017 Feb 10;11:34. doi: 10.3389/fnhum.2017.00034 (PMC5300994; doi:10.3389/fnhum.2017.00034)
Supplement: Supplementary file 2 [file Image_2.pdf]

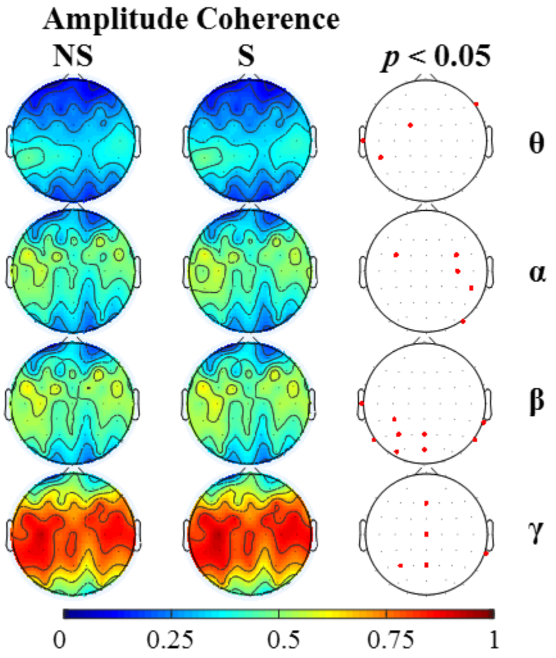

Figure S2. The 2 left columns: for each of the 4 frequency bands [theta (θ), alpha (α), beta (β), gamma (γ)], scalp topographical maps showing location distributions of amplitude coherence between the EEG to the mixed-speech complex and the EEG to a single speech under either the selective attention (S) condition or the non-selective attention (NS) condition. The right columns: for each of the 4 frequency bands, the recordings sites at which the correlation difference between the 2 attention conditions was significant when the p level was 0.05. The statistically thresholded topographical map indicating the more coherence at selective attention condition than non-selective condition in each band. The amplitude coherence is calculated as:

$$Amplitude\ Coherence_{ij} = \frac{\sqrt{\frac{(sA_{ij}^2 - aA_{ij}^2) + (mA_{ij}^2 - aA_{ij}^2)}{2}}}{aA_{ij}^2}$$

where A_{ij} is the Amplitude at the frequency bin I and temporal bin j, respectively. And sA is the amplitude of EEG to the single-speech, mA is the amplitude of EEG to the mixed-speech, and aA is the average of sA and mA.
